# Adverse childhood experiences, lifestyle-related risks, and suicidal ideation and suicide attempts among Chinese college students: joint associations and exploratory network analysis

**DOI:** 10.3389/fpubh.2026.1978715

**Published:** 2026-10-23

**Authors:** Xue Wang, Lei Zhang, Zhe Wang, Xiaoxiao Yuan, Huaning Wang, Mingyang Wu, Wenhua Wang

**Affiliations:** 1 School of General Medicine Xi’an Medical University, Xi’an, China; 2 Shaanxi Provincial Health Industry Association Service Center, Xi’an, China; 3 Shaanxi Medical Association, Xi’an, China; 4 Department of Psychiatry, Xijing Hospital, Air Force Medical University, Xi’an, China; 5 Department of Maternal and Child Health, Xiangya School of Public Health, Central South University, Changsha, China

**Keywords:** additive interaction, adverse childhood experiences, college students, lifestyle-related risks, network analysis, suicidal ideation, suicidality, suicide attempts

## Abstract

**Background:**

Adverse childhood experiences (ACEs) are established correlates of suicidality, but their joint associations with cumulative lifestyle-related risks during emerging adulthood and the conditional connections among specific indicators remain insufficiently characterized.

**Methods:**

A cross-sectional survey was conducted among 18,723 college students selected through multistage cluster sampling from six colleges in Shaanxi Province, China. Thirteen ACE domains were assessed using the Adverse Childhood Experiences International Questionnaire. A cumulative lifestyle-related risk score included smoking, drinking, inadequate physical activity, unhealthy body mass index, and unhealthy diet. Suicidal ideation (SI) and suicide attempt (SA) during the past 12 months were self-reported. Modified Poisson regression was used to estimate adjusted prevalence ratios (*aPRs*). Departure from additivity on the prevalence-ratio scale was evaluated using the relative excess risk due to interaction (*RERI*) and attributable proportion (*AP*). Exploratory network models were used to characterize conditional associations and cross-community connectivity.

**Results:**

The prevalence of SI and SA was 3.3 and 1.9% during the past 12 months, respectively. Greater ACE exposure and higher cumulative lifestyle-related risk score were associated with a higher prevalence of both outcomes. Compared with participants reporting 0–3 ACEs and 0–1 lifestyle-related risk factors, those reporting ≥4 ACEs and ≥2 lifestyle-related risks had the highest adjusted prevalence ratios of SI (*aPR*, 8.37; *95% CI*, 5.64–11.10) and SA (*aPR*, 10.30; *95% CI,* 5.76–14.85). Positive departure from additivity was observed for SI (*RERI*, 3.35; *95% CI*, 1.03–5.97; *AP*, 0.40; *95% CI*, 0.13–0.62) and SA (*RERI*, 5.18; *95% CI*, 1.92–9.78; *AP*, 0.50; *95% CI*, 0.19–0.75). In the exploratory networks, poor sleep showed high cross-community connectivity, while emotional abuse, bullying, inadequate physical activity, and drinking also showed prominent bridge expected influence.

**Conclusion:**

Greater ACE exposure and cumulative lifestyle-related risks were separately and jointly associated with a higher prevalence of SI and SA. Participants reporting both ≥ 4 ACEs and ≥2 lifestyle-related risk profiles had the highest prevalence for SI and SA with evidence of additive interactions. The network results describe cross-sectional conditional associations and should be regarded as hypothesis-generating rather than as evidence of causal or mediating pathways.

## 1 Introduction

Suicide is the second leading cause of death among young adults worldwide (1). Adverse childhood experiences (ACEs), including abuse, neglect, household dysfunction and community adversity, are well-established distal risk factors for suicidal ideation (SI) and suicide attempt (SA) (2–6). However, evidence specific to college students remains limited. Emerging adulthood involves increased autonomy and reduced parental oversight, during which vulnerabilities related to childhood adversity may intensify and manifest as lifestyle-related risk behaviours (7–11). As potential behavioural expressions of maladaptive coping, these factors may represent proximal correlates or potential markers of maladaptive coping among students with prior adversity, but their joint association with ACEs and suicidality remains unclear.

Lifestyle-related risk behaviors are associated with a wide range of adverse health outcomes and have increasingly been linked to depression, psychological distress, and suicidal behaviors (12–17). Compared with adverse childhood experiences (ACEs), lifestyle-related risk behaviors are more proximal and potentially modifiable, and may therefore be relevant to future prevention research among college students. However, whether lifestyle-related risk profiles and ACEs are jointly associated with suicidality in this population remains poorly understood. Several factors may account for this gap. First, lifestyle-related risk behaviors rarely occur in isolation. Smoking, alcohol use, physical inactivity, unhealthy weight, and poor diet often co-occur and may reflect broader behavioural dysregulation, and may be associated with psychological distress and suicide risk (18–20). Analyses focused on single behaviors may underestimate the cumulative burden associated with multiple lifestyle-related risks. Second, ACEs may increase vulnerability to these behavioural patterns by disrupting emotion regulation, stress responsivity, and reward processing, which can promote maladaptive coping behaviors later in life (7, 10, 21–25). Although emerging longitudinal evidence suggests that healthier lifestyle patterns may be associated with more favorable mental health outcomes among individuals exposed to childhood adversity (26–28). However, whether the accumulation of lifestyle-related risks is associated with a higher prevalence of suicidality among college students with ACE exposure remains insufficiently understood. Evidence from adult populations suggests that ACEs are associated with depressive symptoms and other adverse health outcomes, and that lifestyle-related factors may partly account for some of these associations (29, 30). However, whether these findings extend to suicidality among college students remains unclear. In older adults, lifestyle behaviors are often relatively stable, whereas college represents a critical developmental period during which unhealthy behaviors may emerge, cluster, and become established. Examining the joint associations of ACEs and cumulative lifestyle-related risks with suicidality during this stage may therefore help identify modifiable, hypothesis-generating targets for early suicide prevention.

Although cumulative indices are useful for quantifying the overall burden of ACEs and lifestyle-related risk factors, they may obscure substantial heterogeneity across specific ACE domains and individual lifestyle-related risk behaviors (31). Network analysis can estimate conditional associations and identify bridge nodes connecting ACEs, lifestyle-related risk behaviours, and suicidality (32). Importantly, ACEs are distal exposures, whereas lifestyle-related risks are more proximal; their inclusion in the same network does not imply temporal or causal equivalence. Although lifestyle factors may lie on pathways linking early adversity to suicidality, such mediation cannot be established using cross-sectional data. The network analysis was therefore intended not to test mediation, but to reveal cross-domain connectivity that cumulative indices may obscure and to generate hypotheses for future risk assessment and prevention research.

Cumulative indices summarize the overall burden of ACEs and lifestyle-related risks but treat heterogeneous indicators as largely interchangeable and may obscure both the excess prevalence associated with their co-occurrence and the specific factors underlying cross-domain connectivity. To address these limitations, we integrated two complementary analytical approaches. Additive interaction analysis assesses whether the joint prevalence association of ACEs and lifestyle-related risks exceeds the sum of their separate excess associations, thereby quantifying the additional burden associated with their co-occurrence. Exploratory network analysis disaggregates the cumulative indices to characterize conditional associations among individual indicators and identify potential bridge nodes connecting ACEs, lifestyle-related risks, and suicidality. Thus, whereas cumulative indices quantify overall risk burden, additive interaction analysis evaluates excess joint prevalence and network analysis reveals indicator-specific connectivity. Using these complementary approaches, we examined among Chinese college students: (1) the associations of cumulative and domain-specific ACEs and lifestyle-related risks with SI and SA, before and after mutual adjustment; (2) their joint associations and departure from additivity on the prevalence-ratio scale; and (3) the conditional association structure and potential bridge nodes linking specific ACEs, lifestyle-related risks, covariates, and suicidality.

## 2 Methods

### 2.1 Participants

A multistage cluster-sampled cross-sectional survey was conducted among college students in Shaanxi Province, Northwest China in October and November 2022. Six of the 57 eligible colleges in the province were randomly selected. Within participating colleges, 559 classes were sampled proportionally across academic years, and all students enrolled in the selected classes were invited to participate. Two student representatives from each selected class received standardized instructions and distributed the survey link but were not involved in participants’ responses. Participants independently completed the anonymous online questionnaire using their own electronic devices. Participation was voluntary, and informed consent was obtained from all participants, all survey items were mandatory to minimize missing data, participants could withdraw at any time before submitting the questionnaire. Contact information for university mental health services was provided at the end of the survey if needed. Of 20,165 students invited, 19,622 submitted questionnaires. We excluded 612 questionnaires completed in less than 500 s and 287 with prespecified internal inconsistencies, leaving 18,723 participants in the final analytic sample (Figure 1). The study was approved by the Ethics Committee of the Second Affiliated Hospital of Xi’an Jiaotong University (approval number 2022-248) and was conducted in strict accordance with the Declaration of Helsinki.

**Figure 1:**
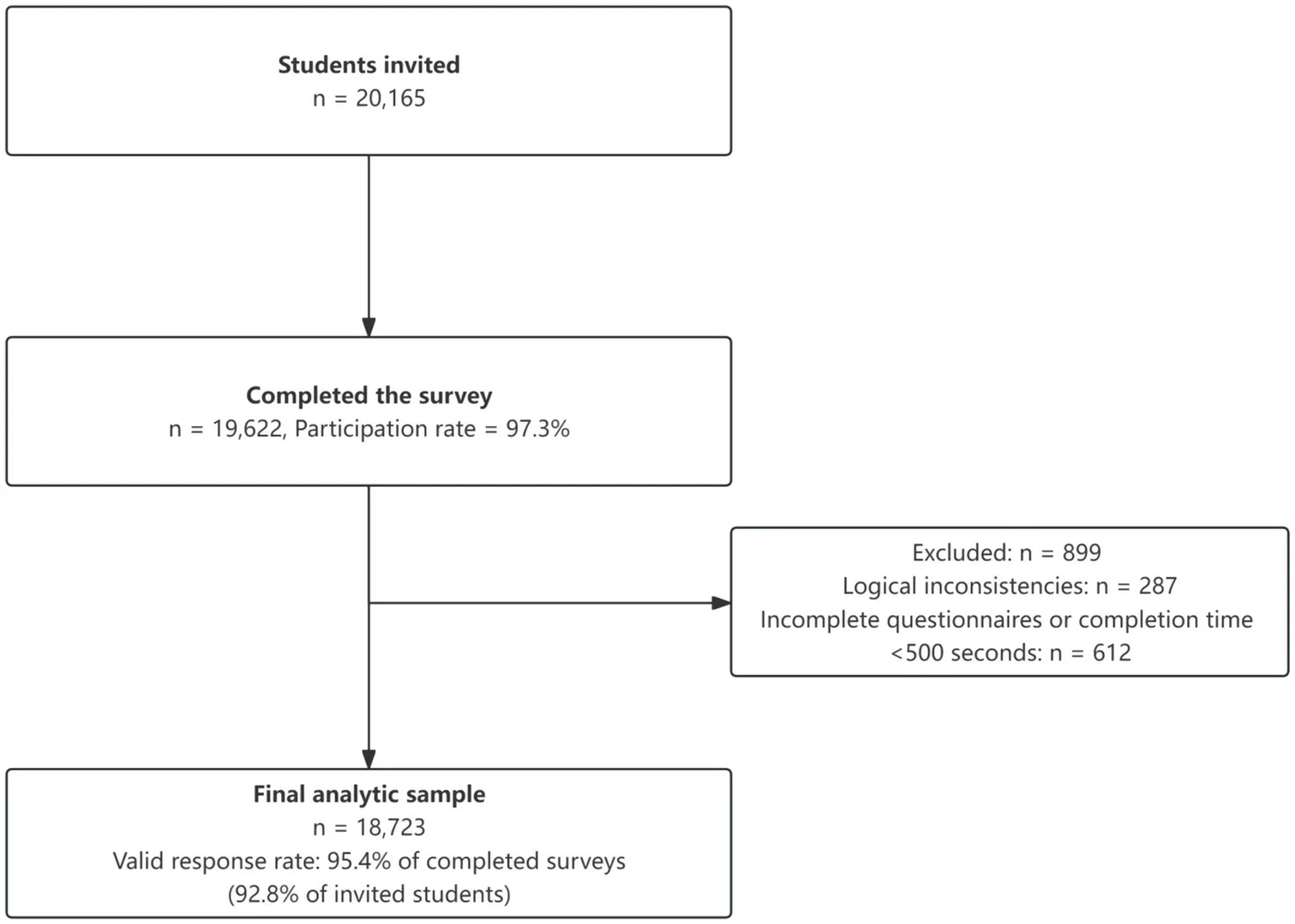
Flowchart.

### 2.2 Measures

#### 2.2.1 Adverse childhood experiences

Adverse childhood experiences were assessed using the Adverse Childhood Experiences International Questionnaire (ACE-IQ) (33). This tool captures 13 diverse domains of early adversity, categorized into four conceptual categories including: abuse (physical, emotional, and sexual), neglect (physical and emotional), household dysfunction (substance abuse, mental illness, incarceration, domestic violence, and parental loss/separation), and peer and community violence (bullying, community, and collective violence). A cumulative ACE score was calculated by assigning one point for each endorsed domain. Consistent with established cut-offs in the literature (34, 35), participants were categorized as having low (0–3 domains) or high (≥4 domains) ACE exposure. As a sensitivity analysis, we repeated the joint-association analyses using an alternative cutoff using low ACE exposure as 0–1 domains and high ACE exposure as ≥2 domains.

#### 2.2.2 Suicidality

Suicidality was assessed using two indicators including suicidal ideation (SI) and suicide attempt (SA) during the past 12 months. SI was assessed by “During the past 12 months, have you ever seriously considered suicide?” SA was measured by “During the past 12 months, did you attempt suicide?” Responses were coded as binary indicators, affirmative responses were classified as SI or SA, respectively (36).

#### 2.2.3 Lifestyle-related risks

We assessed five lifestyle-related risk factors associated with suicidality: current smoking, current drinking, inadequate physical activity, unhealthy body mass index (BMI), and unhealthy diet. Current smoking and drinking were defined as any tobacco or alcohol use within the past 30 days, respectively. Inadequate physical activity was evaluated using the International Physical Activity Questionnaire Short Form (IPAQ-SF), participants who did not meet the criteria for moderate or high metabolic equivalent (MET) category were classified as having inadequate activity levels (37). BMI was calculated as weight in kilograms divided by height in metres squared. Underweight was defined as BMI < 18.5 kg/m^2^ and obesity as BMI ≥ 28.0 kg/m^2^, in accordance with Chinese criteria (38). Participants meeting either criterion were classified as having an unhealthy BMI. Unhealthy diet was defined as daily red meat consumption or less-than-daily intake of fruits and vegetables. A composite unhealthy lifestyle score (ranging from 0–5) was calculated by assigning one point for the presence of each of the five risk factors (Supplementary Table S1). In accordance with established cut-off values from prior literature (39), we stratified participants into two lifestyle-related risk groups as lower lifestyle-risk group (0–1 points) and higher lifestyle-risk group (≥2 points), based on their predefined cumulative unhealthy lifestyle scores. We also assessed lower lifestyle-related risks (0–2) and higher lifestyle-related risks group (≥3 points) in joint association analysis as sensitivity analysis.

#### 2.2.4 Covariates

We adjusted for prespecified sociodemographic covariates selected from prior literature: sex (male or female), grade (1st, 2nd, 3rd, or 4th+), ethnicity (Han or minority), residence type (rural or urban), sibling status (only child or having siblings), maternal educational attainment, and paternal educational attainment (middle school or below, high school, or college or above). Depressive symptoms, sleep quality, and social support may be confounders or intermediates, including them in the primary models could result in overadjustment, so they were included in conservative sensitivity analyses. Depressive symptoms were assessed using a modified 19-item version of Zung Self-Rating Depression Scale (SDS). Item 19 “I feel that others would be better off if I were dead” was excluded to avoid conceptual overlap with the suicidality outcomes. The total score yield 19 to 76, with a standardized score ≥48.75 (equivalent to a raw SDS-19 total score ≥39) indicating elevated depressive symptoms (40). The 20-item SDS showed Cronbach’s *α* = 0.843 and McDonald’s *ω* = 0.878; after excluding Item 19, the modified 19-item SDS showed *α* = 0.842 and *ω* = 0.879. Poor sleep was assessed with the Pittsburgh Sleep Quality Index (PSQI), yielding a global score ranging from 0 to 21, using a validated cutoff of ≥8 (41). The PSQI item set used to calculate the global score showed *α* = 0.849 and *ω* = 0.850. Social support was assessed using the 17-item Social Support Scale for University Students developed by Ye (42), yielding a total score ranging from 17 to 85, analyzed as a continuous variable in regression models and dichotomized at the sample median (67) for network analysis In the current sample, Cronbach’s *α* was 0.980 and McDonald’s *ω* was 0.980. A one-factor CFA using WLSMV in an independent random half-sample (*n* = 9,362) yielded CFI = 0.998, TLI = 0.997, RMSEA = 0.135 (90% CI, 0.134–0.137), and SRMR = 0.039. The fit indices were mixed: CFI, TLI, and SRMR indicated favorable fit, whereas RMSEA indicated poor fit. These findings therefore do not provide unequivocal support for a strictly unidimensional structure.

#### 2.2.5 Statistical analysis

Continuous variables were summarized as means and standard deviations (*SD*), and categorical variables as frequencies and percentages (*n* [%]). Group differences were compared with t tests or chi-square tests as appropriate. Modified Poisson regression with robust variance estimation was used to estimate prevalence ratios (PRs) and 95% confidence intervals (CIs) for the associations of ACEs and lifestyle-related risks with SI and SA. The sandwich variance was clustered by the school-by-class response clusters. Four models were constructed. Model 1 was unadjusted. Model 2 adjusted for sex, grade, ethnicity, residence type, sibling status, and maternal and paternal educational attainment. Model 3 additionally included mutual adjustment for cumulative ACEs and cumulative lifestyle-related risks. Because depressive symptoms, poor sleep, and social support could be confounders, correlates, or consequences of the exposures, they were included only in an exploratory sensitivity model (Model 4). For descriptive analyses, ACEs were categorized as 0, 1, 2–3, and ≥4 domains, and cumulative lifestyle-risk score was categorized as 0–1, 2, and 3–5 factors. The scores were also analyzed continuously to estimate the PR associated with each additional ACE domain or lifestyle-related risk factor. For joint-association analyses, participants were cross-classified into four mutually exclusive groups using ACE exposure of 0–3 versus ≥4 domains and lifestyle-related risk scores of 0–1 versus ≥2. Covariate-standardized prevalence estimates and prevalence differences (PDs) were obtained from Model 3 using marginal standardization. The method used to calculate their 95% CIs should be specified. We obtained covariate-standardized prevalences for the four groups from the Model 3 design matrix. We performed two sets of sensitivity analyses for the cumulative and joint-association analyses. First, we examined alternative exposure cutoffs by dichotomizing ACEs as 0–1 versus ≥2 and lifestyle-related risks as 0–2 versus ≥3. Second, to assess whether the exclusion of poor sleep from the primary lifestyle-risk score affected the findings, we reconstructed the score by including poor sleep as a sixth lifestyle-related risk indicator. The resulting score ranged from 0 to 6 and was categorized as 0–1, 2, and 3–6 risks for the cumulative-risk analysis. For the corresponding joint-association analysis, lifestyle-related risks were classified as lower (0–1) versus higher (≥2), while ACE exposure was classified as 0–3 versus ≥4. In these analyses, poor sleep was treated as an exposure component and was therefore not entered separately as a covariate. Results are presented in Supplementary Tables S6–S9. Departure from additivity on the prevalence-ratio scale was evaluated using the relative excess risk due to interaction (RERI) and attributable proportion (AP): *RERI = PR_11_-PR_10_-PR_01_ + 1*. And AP was calculated as: *AP = RERI/PR_11_*. Where *PR_11_* denotes joint exposure to high ACEs and high lifestyle-related risks, whereas *PR_10_* and *PR_01_* denote high ACE exposure only and high lifestyle-related risks only, respectively. Confidence intervals were estimated using 1,000 bootstrap resamples. Because the sample was cluster-based, bootstrap resampling was conducted at the class level. A *95% CI* for *RERI* or *AP* that excluded zero was interpreted as evidence of departure from additivity in this sample. These measures were treated as descriptive interaction contrasts rather than causal interaction estimates. We estimated two separate 29-node networks in the full sample, one for SI and one for SA. Each network included 13 ACE domains, five lifestyle-related risk factors, one suicidality indicator, and 10 prespecified sociodemographic and psychosocial covariates. All nodes were binary coded, with definitions and coding details provided in Supplementary Table S10. Accordingly, pairwise binary Ising models were estimated using the IsingFit package with L1-regularized nodewise logistic regression and extended Bayesian information criterion (EBIC) model selection (*γ* = 0.50). Edges represent regularized pairwise conditional associations after accounting for all other nodes. We assessed node strength, expected influence, bridge expected influence across four prespecified communities (ACEs, lifestyle-related risks, suicidality, and covariates), and predictability using classification accuracy. Edge-weight accuracy and centrality stability were evaluated using nonparametric and case-dropping bootstrapping, respectively, with 1,000 resamples each. Given the cross-sectional design and differing temporal reference periods of the nodes, the networks were intended solely to characterize conditional dependence. Thus, edges should not be interpreted as causal effects, temporal or developmental pathways, mediating mechanisms, or evidence that one node preceded another. We conducted all statistical analyses using R version 4.0.2. Network models were constructed with the bootnet, qgraph, networktools, and mgm packages. All tests were two-sided, and statistical significance was defined as *p* < 0.05.

## 3 Results

### 3.1 Sample characteristics

Among 18,723 participants, 619 (3.3%) reported SI and 362 (1.9%) reported SA during the past 12 months (Supplementary Table S2). Most participants were female (65.1%), of Han ethnicity (97.0%), and rural residence (54.0%). SI differed significantly by grade, residence type, and paternal education, while SA differed by residence type (all *p* < 0.001). Participants with SI or SA had higher cumulative lifestyle-related risk factors and greater ACE exposure (all *p* < 0.001). Detailed distributions of ACEs and lifestyle factors are presented in Supplementary Tables S3, S4.

### 3.2 Associations of ACEs with suicidality

In Model 3, each additional ACE dimension was associated with higher prevalence of SI (*aPR*, 1.32; *95% CI*, 1.29–1.35) and SA (*aPR*, 1.33; *95% CI*, 1.29–1.36). Compared with participants reporting no ACEs, those reporting 1, 2–3, and ≥4 domains had progressively higher prevalence of SI 1.80 (*95% CI*, 1.24–2.61), 4.95 (*95% CI*, 3.33–7.36), 11.71 (*95% CI*, 8.02–17.10) and SA 2.81 (*95% CI*, 1.64–4.80), 5.32 (*95% CI*, 3.03–9.33), 14.78 (*95% CI*, 8.59–25.44), respectively. All four ACE categories were positively associated with both SI and SA in Model 3, with the largest point estimates observed for abuse and peer/community adversity. Associations were attenuated after further adjustment for poor sleep, depressive symptoms, and social support (Model 4) but generally remained significant. Each additional ACE dimension remained associated with SI (*aPR*, 1.22; *95% CI*, 1.19–1.25) and SA (*aPR*, 1.23; *95% CI*, 1.19–1.27), whereas the associations of neglect with SI and SA were no longer statistically significant. Among individual ACE dimensions, sexual abuse showed the highest prevalence ratios with SI and household mental illness with SA (Supplementary Table S5; Table 1).

**Table 1:** Associations of cumulative ACE exposure and broad ACE categories with SI and SA.

Variables	Suicidal ideation (SI), aPR (95% CI)				Suicide attempt (SA), aPR (95% CI)			
	Model 1	Model 2	Model 3	Model 4	Model 1	Model 2	Model 3	Model 4
Cumulative ACEs, per one-domain increase	1.32 (1.30–1.35)	1.33 (1.30–1.35)	1.32 (1.29–1.35)	1.22 (1.19–1.25)	1.33 (1.30–1.37)	1.34 (1.30–1.37)	1.33 (1.29–1.36)	1.23 (1.19–1.27)
Number of ACE domains								
0(reference)	Reference	Reference	Reference	Reference	Reference	Reference	Reference	Reference
1	1.85 (1.28–2.68)	1.87 (1.29–2.72)	1.80 (1.24–2.61)	1.45 (1.00–2.10)	2.95 (1.72–5.07)	2.99 (1.74–5.13)	2.81 (1.64–4.80)	2.17 (1.27–3.71)
2–3	5.10 (3.43–7.59)	5.23 (3.52–7.77)	4.95 (3.33–7.36)	3.03 (2.03–4.52)	5.69 (3.24–9.99)	5.79 (3.30–10.17)	5.32 (3.03–9.33)	3.04 (1.74–5.32)
≥4	12.11 (8.30–17.66)	12.63 (8.67–18.41)	11.71 (8.02–17.10)	5.05 (3.43–7.44)	16.09 (9.35–27.70)	16.60 (9.64–28.59)	14.78 (8.59–25.44)	5.83 (3.36–10.12)
Broad ACE categories								
abuse (vs. No)	4.19 (3.60–4.89)	4.27 (3.66–4.97)	4.14 (3.55–4.83)	2.58 (2.21–3.02)	3.97 (3.21–4.92)	4.01 (3.24–4.96)	3.83 (3.09–4.74)	2.31 (1.86–2.86)
neglect (vs. No)	1.39 (1.16–1.65)	1.41 (1.18–1.68)	1.37 (1.15–1.64)	1.04 (0.87–1.23)	1.68 (1.32–2.13)	1.70 (1.34–2.17)	1.65 (1.30–2.09)	1.24 (0.97–1.58)
household dysfunction (vs. No)	3.46 (2.95–4.05)	3.48 (2.97–4.08)	3.37 (2.88–3.96)	2.21 (1.88–2.61)	3.50 (2.84–4.31)	3.51 (2.84–4.33)	3.35 (2.72–4.13)	2.14 (1.73–2.65)
peer/community adversity (vs. No)	3.54 (3.05–4.11)	3.67 (3.15–4.26)	3.55 (3.05–4.13)	2.25 (1.93–2.62)	3.22 (2.60–3.97)	3.30 (2.67–4.08)	3.15 (2.54–3.89)	1.93 (1.56–2.39)

ACEs, adverse childhood experiences. aPR, adjusted prevalence ratio; CI, confidence interval. Model 1 is unadjusted. Model 2 is adjusted for sex, grade, ethnicity, residence type, sibling status, maternal educational attainment and paternal educational attainment. Model 3 is adjusted for sex, grade, ethnicity, residence type, sibling status, maternal educational attainment, paternal educational attainment, and cumulative lifestyle-related risk factor. Each broad ACE category was entered in a separate model. Model 4 additionally adjusts for poor sleep, depressive symptoms, and social support.

### 3.3 Associations of lifestyle-related risks with suicidality

In Model 3, each additional lifestyle-related risk factor was associated with a higher prevalence of SI (*aPR*, 1.34; 95% CI, 1.23–1.45) and SA (*aPR*, 1.46; *95% CI*, 1.32–1.61). Compared with participants reporting 0–1 lifestyle-related risk factors, those reporting 3–5 factors had higher prevalence of SI (*aPR*, 1.91; *95% CI*, 1.45–2.52) and SA (*aPR*, 2.66; *95% CI*, 1.84–3.85). Smoking, drinking, inadequate physical activity, and unhealthy diet were positively associated with SI and SA, whereas unhealthy BMI was not statistically significant. Associations were attenuated in Model 4, but cumulative lifestyle-related risks, smoking, and drinking remained associated with both outcomes; inadequate physical activity remained associated only with SA (Table 2).

**Table 2:** Associations of cumulative and specific lifestyle-related risk factors with SI and SA.

Variables	Suicidal ideation (SI), aPR (95% CI)				Suicide attempt (SA), aPR (95% CI)			
	Model 1	Model 2	Model 3	Model 4	Model 1	Model 2	Model 3	Model 4
Cumulative lifestyle-related risk score, per one-factor increase	1.46 (1.34–1.60)	1.48 (1.35–1.62)	1.34 (1.23–1.45)	1.17 (1.08–1.27)	1.60 (1.44–1.78)	1.62 (1.45–1.79)	1.46 (1.32–1.61)	1.25 (1.13–1.38)
Lifestyle-related risk profiles								
0-1(reference)	Reference	Reference	Reference	Reference	Reference	Reference	Reference	Reference
2	1.35 (1.02–1.77)	1.38 (1.05–1.82)	1.32 (1.00–1.73)	1.14 (0.88–1.50)	1.51 (1.03–2.23)	1.56 (1.06–2.29)	1.48 (1.01–2.17)	1.23 (0.85–1.80)
3–5	2.22 (1.68–2.92)	2.27 (1.72–2.99)	1.91 (1.45–2.52)	1.42 (1.08–1.86)	3.08 (2.12–4.47)	3.15 (2.17–4.57)	2.66 (1.84–3.85)	1.86 (1.29–2.68)
Specific lifestyle factors (vs. No)								
smoking (vs. No)	1.75 (1.43–2.14)	2.01 (1.62–2.48)	1.57 (1.27–1.94)	1.23 (1.01–1.51)	2.00 (1.55–2.58)	2.26 (1.73–2.95)	1.79 (1.37–2.32)	1.33 (1.03–1.72)
drinking (vs. No)	2.01 (1.70–2.39)	2.07 (1.74–2.47)	1.53 (1.29–1.82)	1.25 (1.05–1.48)	2.19 (1.77–2.70)	2.22 (1.80–2.74)	1.68 (1.37–2.08)	1.31 (1.06–1.61)
inadequate physical activity (vs. No)	1.27 (1.05–1.54)	1.29 (1.06–1.56)	1.36 (1.12–1.64)	1.19 (0.99–1.42)	1.48 (1.14–1.91)	1.52 (1.17–1.97)	1.58 (1.22–2.03)	1.35 (1.05–1.73)
unhealthy BMI (vs. No)	1.20 (1.02–1.41)	1.16 (0.99–1.35)	1.15 (0.99–1.34)	1.11 (0.95–1.29)	1.24 (0.99–1.54)	1.20 (0.97–1.50)	1.19 (0.96–1.48)	1.16 (0.94–1.43)
unhealthy diet (vs. No)	2.07 (1.36–3.13)	2.18 (1.44–3.31)	1.90 (1.26–2.85)	1.49 (1.00–2.20)	2.94 (1.48–5.82)	3.08 (1.56–6.11)	2.67 (1.36–5.25)	1.94 (1.00–3.77)

BMI, body mass index; aPR, adjusted prevalence ratio; CI, confidence interval. Model 1 is unadjusted. Model 2 is adjusted for sex, grade, ethnicity, residence type, sibling status, maternal educational attainment and paternal educational attainment. Model 3 is adjusted for sex, grade, ethnicity, residence type, sibling status, maternal educational attainment, paternal educational attainment, and ACEs domains. Each lifestyle factor was examined in a separate model. Model 4 additionally adjusts for poor sleep, depressive symptoms, and social support.

### 3.4 Joint associations of ACEs and lifestyle-related risks with suicidality

Participants with ≥4 ACE domains and ≥2 lifestyle-related risk factors had the highest adjusted prevalence of SI (13.08%) and SA (7.71%). Compared with those reporting 0–3 ACE domains and 0–1 lifestyle-related risk factors, they had substantially higher prevalence of SI (*aPR*, 8.37; *95% CI*, 5.64–11.10) and SA (*aPR*, 10.30; *95% CI*, 5.76–14.85). Under the primary cutoffs, positive departure from additivity was observed for SI (*RERI*, 3.35; *95% CI*, 1.03–5.97; *AP*, 0.40; *95% CI*, 0.13–0.62) and SA (*RERI*, 5.18; *95% CI*, 1.92–9.78; *AP*, 0.50; *95% CI*, 0.19–0.75). Sensitivity analyses using alternative cutoffs for cumulative ACEs (≥2) and lifestyle-related risks (≥3) the RERI for SI was attenuated and its CI included zero SI (*RERI,*1.27; *95% CI*, −0.04-2.75), whereas positive departure from additivity remained evident for SA (*RERI*, 2.63;*95% CI*, 0.95–4.39) (Supplementary Table S6). In domain-specific analyses, abuse, household dysfunction, and peer/community adversity showed positive additive interactions with lifestyle-related risks for both SI and SA, whereas no clear evidence of additive interaction was observed for neglect (Supplementary Table S7). Sensitivity analyses treating poor sleep as a sixth lifestyle-related risk factor yielded similar findings, showed in Supplementary Table S8, S9 (Table 3).

**Table 3:** Joint associations and additive interactions of cumulative ACEs and lifestyle-related risks with SI and SA.

ACE exposure/Lifestyle-related risk group	Total N	Cases (*n*, %)	Adjusted prevalence, % (95% CI)	aPR (95% CI)	PD, percentage points (95% CI)	RERI (95% CI)	AP (95% CI)
Suicidal ideation (SI)							
ACEs (0–3)							
lower lifestyle-related risk (0–1)	2,996	48 (1.6)	1.56 (1.10–2.02)	Reference	Reference	–	–
higher lifestyle-related risks (≥2)	13,719	332 (2.4)	2.43 (2.16–2.69)	1.55 (1.07–2.04)	0.86 (0.34–1.39)	–	–
ACEs (≥4)							
lower lifestyle-related risk (0–1)	291	20 (6.9)	6.98 (3.96–10.01)	4.47 (2.22–6.72)	5.42 (2.40–8.44)	–	–
higher lifestyle-related risks (≥2)	1717	219 (12.8)	13.08 (11.36–14.80)	8.37 (5.64–11.10)	11.52 (9.72–13.32)	3.35 (1.03–5.97)	0.40 (0.13–0.62)
Suicide attempt (SA)							
ACEs (0–3)							
lower lifestyle-related risk (0–1)	2,996	23 (0.8)	0.75 (0.44–1.05)	Reference	Reference	–	–
higher lifestyle-related risks (≥2)	13,719	200 (1.5)	1.46 (1.25–1.67)	1.96 (1.11–2.80)	0.71 (0.34–1.08)	–	–
ACEs (≥4)							
lower lifestyle-related risk (0–1)	291	9 (3.1)	3.12 (1.11–5.13)	4.17 (0.97–7.37)	2.37 (0.33–4.41)	–	–
higher lifestyle-related risks (≥2)	1717	130 (7.6)	7.71 (6.43–8.99)	10.30 (5.76–14.85)	6.96 (5.65–8.27)	5.18 (1.92–9.78)	0.50 (0.19–0.75)

ACEs, adverse childhood experiences; aPR, adjusted prevalence ratio; CI, confidence interval; PD, prevalence difference; RERI, relative excess risk due to interaction; AP, attributable proportion. Model is adjusted for sex, grade, ethnicity, residence type, sibling status, maternal educational attainment and paternal educational attainment.

### 3.5 Exploratory network analysis

Each network comprised 29 nodes representing one suicidality outcome, 13 ACE domains, five lifestyle factors, and 10 covariates in Figure 2 and Supplementary Figures S1, S2. In the SI network, the largest outcome-related edges were observed for depressive symptoms (0.820), poor sleep (0.677) and emotional abuse (0.644). In the SA network, the largest positive outcome-related edge weights were observed for depressive symptoms (1.004), poor sleep (0.612), domestic violence (0.508) and household mental illness (0.496). In bridge expected influence analyses, poor sleep showed the highest bridge expected influence among non-suicidality nodes in both networks (Figure 3). Emotional abuse and bullying were among ACE nodes with the highest positive bridge expected influence. Among lifestyle factors, inadequate physical activity and drinking were prominent bridge expected influence in the SI and SA networks. Expected-influence, strength and bridge expected influence centrality stability coefficients were 0.750 in both networks in Supplementary Figure S3.

**Figure 2:**
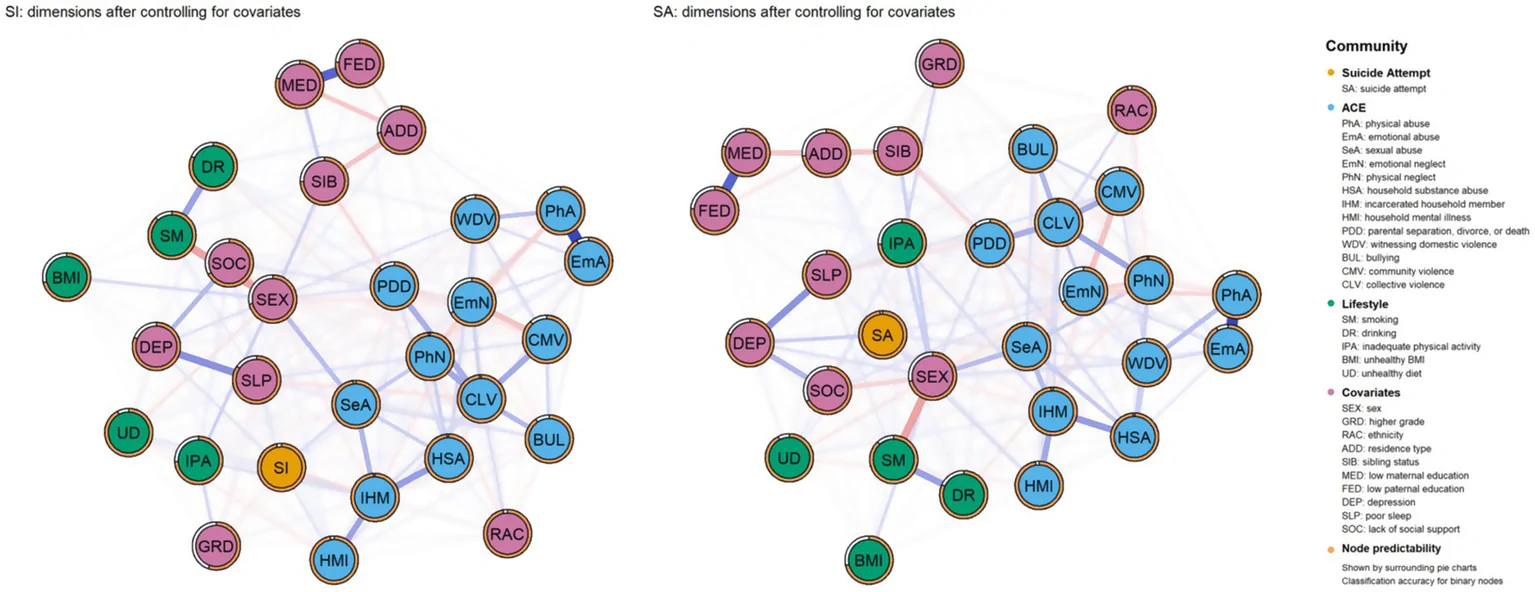
Adjusted dimension-level networks for suicidal ideation and suicide attempt. Two side-by-side network diagrams show regularized conditional associations for SI **(A)** and SA **(B)**. Nodes represent the suicide-related outcome, adverse childhood experience domains, lifestyle-risk factors, and covariates. Lines connecting nodes indicate non-zero conditional associations estimated jointly with the covariate nodes; line type and colour distinguish positive from negative associations, and thicker lines indicate stronger associations. Depressive symptoms have the strongest positive conditional association with each outcome. Rings surrounding the nodes represent predictability, measured as classification accuracy for binary variables.

**Figure 3:**
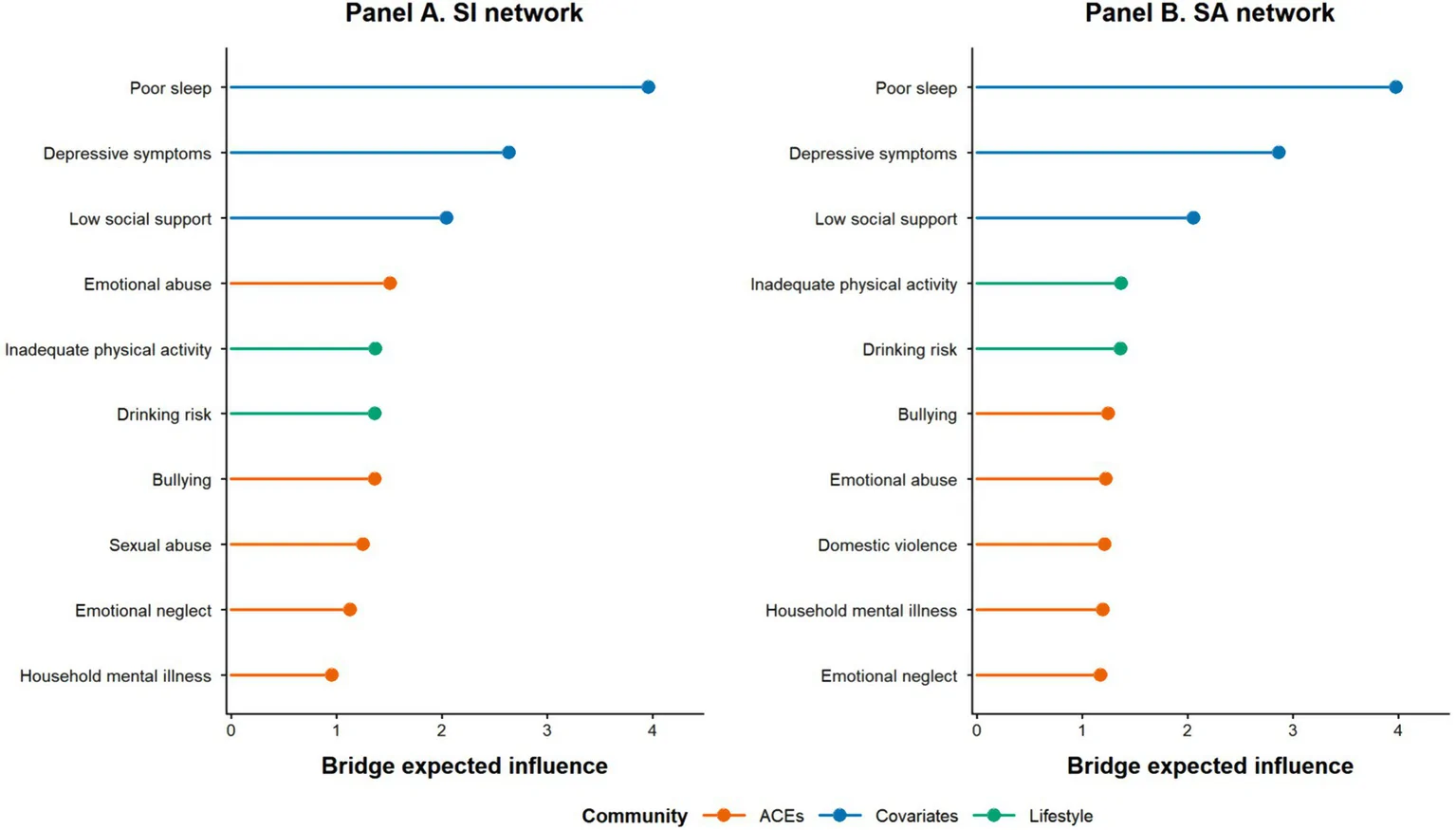
Bridge expected influence in adjusted dimension-level networks. Two ranked plots show bridge expected influence among non-suicidality nodes in the networks including binary covariate nodes for SI **(A)** and SA **(B)**. Bridge expected influence summarizes each node’s conditional connections with nodes in other prespecified communities, comprising ACEs, lifestyle-related risks, covariates, and suicidality; the covariates community included sociodemographic and psychosocial variables. Poor sleep showed high cross-community connectivity under the prespecified network structure. Emotional abuse, bullying, inadequate physical activity and drinking also showed cross-community connectivity. These variables warrant prospective evaluation rather than being interpreted as established intervention targets. SI and SA are excluded from their respective rankings.

## 4 Discussion

In this large cross-sectional study of Chinese college students, greater ACE exposure and higher cumulative lifestyle-related risks were associated with a higher prevalence of SI and SA during the past 12 months. Participants reporting both ≥4 ACE domains and ≥2 lifestyle-related risk factors had the highest aPRs of SI and SA, with positive additive interactions observed. In exploratory conditional association networks, depressive symptoms had the largest positive edge weight with SI and SA, whereas poor sleep showed the highest bridge expected influence. Emotional abuse, bullying, inadequate physical activity, and drinking showed prominent cross-community connectivity. These network results should be viewed as descriptive and hypothesis-generating rather than as evidence of temporal or causal pathways.

The association between ACEs and suicidality in this sample is consistent with evidence from the World Mental Health International College Student (WMH-ICS) initiative and studies conducted across diverse socioeconomic and cultural contexts (9, 11, 43–47). Previous research among Chinese college students has identified coping styles and resilience as potential mediators of the association between childhood unpredictability and depression (48), suggesting that both psychosocial resources and behavioral responses may shape the long-term mental health consequences of adverse childhood environments. Each additional ACE dimension was positively associated with a higher prevalence of both SI and SA, suggesting a graded relationship between cumulative adversity and suicidality during emerging adulthood. This pattern is aligned with prior evidence showing that each additional adverse exposure is associated with a substantially increased likelihood of SI (49). Moreover the graded associations across 0, 1, 2–3 and ≥4 ACE domains support a cumulative-burden pattern, although equal weighting of heterogeneous domains remains a measurement limitation. The associations also varied across ACE categories and individual dimensions. Abuse, household dysfunction, and peer/community adversity were positively associated with both SI and SA, although point estimates tended to be larger for SA than for SI, these differences were not formally tested and therefore should not be interpreted as evidence that specific ACE domains are more strongly associated with SA. Previous research has also demonstrated that combinations of childhood family abuse and peer bullying may have complex, nonlinear associations with adolescent psychological outcomes (50). Threat-related ACEs, including physical and emotional abuse, domestic violence, and bullying, were associated with suicidality, particularly SA, consistent with evidence linking early threat exposure to altered stress processing, emotion regulation, and interpersonal functioning (51–54). However, the associations with neglect should be interpreted cautiously, as they were no longer statistically significant after adjustment for poor sleep, depressive symptoms, and social support. This attenuation may reflect shared variance among co-occurring ACEs, measurement differences, or potential overadjustment for factors that may lie on the pathway between ACEs and suicidality (55).

Greater cumulative lifestyle-related risk factors were associated with a higher prevalence of SI and SA, consistent with studies flinking clustered behavioral risks to suicidality among young people (18–20). hese associations remained after adjustment for ACE exposure, but they should not be interpreted as independent or causal effects. Smoking, drinking, inadequate physical activity, and unhealthy diet may co-occur with psychological distress, depressive symptoms, maladaptive coping, and impaired self-regulation, and reverse or bidirectional associations are possible (14, 15, 17, 56, 57).

Participants with high ACE exposure and cumulative lifestyle-related risks had the highest prevalence of SI and SA. This pattern is consistent with prior evidence that childhood adversity, health-risk behaviour, and self-injurious outcomes frequently co-occur (39). Positive departure from additivity on the prevalence-ratio scale suggested that the joint prevalence association exceeded the sum of the separate excess associations under the primary exposure classifications. However, the SI interaction estimate was attenuated and imprecise when alternative cutoffs were applied, indicating sensitivity to exposure categorization. Previous studies have suggested that healthier lifestyle patterns are associated with better mental health among individuals exposed to childhood adversity, including lower depressive symptoms (27, 28). Nevertheless, the present findings do not demonstrate that lifestyle modification would reduce ACE-related suicidality. Instead, they indicate that students with both high ACE exposure and multiple lifestyle-related risks may constitute a subgroup with a particularly high concurrent prevalence of SI and SA. Given the cross-sectional design and the small numbers of outcomes in some joint-exposure groups, the interaction estimates should be interpreted cautiously and not as evidence of causal interaction.

The exploratory network analyses complemented the cumulative and interaction analyses by identifying conditional associations among specific ACE dimensions, lifestyle-related risk factors, and suicidality in college students. Depressive symptoms showed the strongest conditional association with both SI and SA, consistent with their established clinical relevance to suicidal thoughts and behaviours (58). Among ACE domains, emotional abuse and bullying showed the largest edges weight with SI, whereas witnessing domestic violence and household mental illness showed largest weight with SA. This pattern highlights interpersonal victimisation and household dysfunction as potentially important correlates of suicidality after accounting for co-occurring adversities and behavioural factors. Emotional abuse and bullying were prominent ACE bridge nodes, indicating that they may connect ACEs with lifestyle-related risks and suicidality across network communities. One possible interpretation is that threat-related adversities, including emotional and physical abuse, witnessing domestic violence, and bullying, may be linked to suicidality through heightened threat sensitivity, dysregulated stress responses, emotion-regulation difficulties, and maladaptive interpersonal expectations (59–61). In contrast, deprivation-related experiences may operate through reduced social and cognitive resources, insecure attachment, and lower perceived support. These mechanisms were not directly tested and therefore remain hypothetical.

Among lifestyle factors, inadequate physical activity and drinking were prominent bridge nodes in the SI and SA networks. These findings identify variables that warrant prospective investigation but do not establish that they mediate the association between ACEs and suicidality or represent effective intervention targets. From an assessment perspective, considering specific interpersonal and household adversities alongside depressive symptoms, sleep problems, and behavioral factors may provide a broader clinical profile of students reporting SI or SA. By including ACEs and more proximal lifestyle-related risks in the same cross-sectional network, this study identified indicator-specific and cross-domain conditional associations that cumulative indices may obscure. However, the model could not establish their temporal ordering or developmental pathways. Longitudinal studies with repeated assessments are needed to examine whether lifestyle-related risks mediate or otherwise contribute to the association between ACEs and subsequent suicidality.

### 4.1 Limitations

Several limitations should be considered. First, the cross-sectional design precludes temporal or causal inference. Although ACEs refer to childhood exposures, they were assessed retrospectively, and lifestyle-related risk factors, depressive symptoms, poor sleep, social support, and suicide-related outcomes were measured concurrently. Reverse and bidirectional associations therefore remain possible. Second, reliance on self-reported measures may introduce recall, reporting, and common-method biases. Retrospective self-reported ACEs may be influenced by current psychological distress, stigma, and underreporting. Longitudinal evidence among Chinese adolescents indicates that depressive symptoms may predict greater subsequent recall of ACEs, highlighting the possibility of mood-related autobiographical memory bias (62). Consequently, differential recall among participants with greater psychological distress or suicidality may have affected the estimated associations. Third, the cumulative ACE and lifestyle-related risk indices assigned equal weight to heterogeneous indicators and did not capture their severity, frequency, duration, or developmental timing. Categorization also resulted in information loss, and the additive interaction estimates showed some sensitivity to the selected thresholds. Fourth, potentially relevant lifestyle-related risks, including problematic internet use, screen time, and irregular sleep schedules, were not comprehensively assessed, leaving the possibility of residual confounding or incomplete characterization of lifestyle risk. Fifth, SI and especially SA were relatively uncommon, and some joint-exposure groups included few cases, reducing statistical precision. Sixth, participants were sampled from six colleges in a single Chinese province and should not be considered nationally representative, which may limit generalizability to other regions, non-college emerging adults, and different cultural or educational settings. Finally, network estimates depend on node selection, measurement quality, community definition, regularization, and model specification. Network edges and bridge centrality represent conditional associations rather than ausal effects, temporal pathways, mediating mechanisms, or intervention relevance. Moreover, the SI and SA networks were not formally compared, so apparent differences between them should be interpreted cautiously.

## 5 Conclusion

Greater ACE exposure and higher cumulative lifestyle-related risk scores were associated with a higher past-12-month prevalence of SI and SA among Chinese college students. Students reporting both ≥4 ACE domains and ≥2 lifestyle-related risk factors had the highest concurrent prevalence of both outcomes, with evidence of positive departure from additivity under the primary exposure classifications. Exploratory network analyses identified depressive symptoms, poor sleep, emotional abuse, bullying, inadequate physical activity, and drinking as nodes with prominent conditional or cross-community connectivity. These cross-sectional findings are hypothesis-generating and require replication using longitudinal designs before temporal, causal, mediating, or intervention-related conclusions can be drawn.

## Data Availability

The data analyzed in this study is subject to the following licenses/restrictions: de-identified data may be available from the corresponding author on reasonable request and with permission from the relevant ethics committee and participating institutions. Requests to access these datasets should be directed to 534154497@qq.com.
