# Supplementary material for: Adverse childhood experiences, lifestyle-related risks, and suicidal ideation and suicide attempts among Chinese college students: joint associations and exploratory network analysis
Source: Front Public Health. 2026 Oct 23;14:1978715. doi: 10.3389/fpubh.2026.1978715 (PMC13741368; doi:10.3389/fpubh.2026.1978715)
Supplement: Supplementary file 1 [file Table_1.DOCX]

**Supplementary Table S1. Definitions of unhealthy lifestyle factors**

| Unhealthy lifestyle factors | Definition |
| --- | --- |
| Smoking | Any tobacco use during the past 30 days |
| Drinking | Any alcohol use during the past 30 days |
| Unhealthy diet | Any of the following 3 characteristics, as assessed by food frequency questionnaire 1.Fruits: <7times/week 2.Vegetables: <7times/week 3.Red meat: ≥7times/week |
| Inadequate physical activity | participants who did not meet the criteria for moderate or high physical activity according to IPAQ-SF |
| Unhealthy BMI | BMI≥28 kg/m^2^ or BMI<18.5 kg/m^2^ |

**Supplementary Table S2. Characteristics of participants according to suicidal ideation and suicide attempt status (N = 18,723)**

| Characteristics | **Suicidal Ideation (SI)** | | P | **Suicide Attempt (SA)** | | P |
| --- | --- | --- | --- | --- | --- | --- |
|  | Never | Ever |  | Never | Ever |  |
| **Sex, n (%)** |  |  | 0.013 |  |  | 0.276 |
| Male | 6344 (35.0) | 187 (30.2) |  | 6415 (34.9) | 116 (32.0) |  |
| Female | 11,760 (65.0) | 432 (69.8) |  | 11,946 (65.1) | 246 (68.0) |  |
| **Grade, n (%)** |  |  | **<0.001** |  |  | 0.068 |
| 1st | 5261 (29.1) | 192 (31.0) |  | 5336 (29.1) | 117 (32.3) |  |
| 2nd | 4294 (23.7) | 174 (28.1) |  | 4371 (23.8) | 97 (26.8) |  |
| 3rd | 4250 (23.5) | 149 (24.1) |  | 4317 (23.5) | 82 (22.7) |  |
| 4th+ | 4299 (23.7) | 104 (16.8) |  | 4337 (23.6) | 66 (18.2) |  |
| **Ethnicity, n (%)** |  |  | 0.167 |  |  | 0.800 |
| Han | 17,575 (97.1) | 595 (96.1) |  | 17,820(97.1) | 350 (96.7) |  |
| others | 529 (2.9) | 24 (3.9) |  | 541 (2.9) | 12 (3.3) |  |
| **Residence type, n (%)** |  |  | **<0.001** |  |  | **<0.001** |
| Rural | 9834 (54.3) | 283 (45.7) |  | 9954 (54.2) | 163 (45.0) |  |
| Urban | 8270 (45.7) | 336 (54.3) |  | 8407 (45.8) | 199 (55.0) |  |
| **Sibling status, n (%)** |  |  | 0.057 |  |  | 0.217 |
| Only child | 5325 (29.4) | 204 (33.0) |  | 5411 (29.5) | 118 (32.6) |  |
| Having sibling(s) | 12,779 (70.6) | 415 (67.0) |  | 12,950(70.5) | 244 (67.4) |  |
| **Maternal educational attainment, n (%)** |  |  | **<0.001** |  |  | 0.047 |
| Middle school or below | 11,687 (64.6) | 353 (57.0) |  | 11,821(64.4) | 219 (60.5) |  |
| High school | 3635 (20.1) | 143 (23.1) |  | 3708 (20.2) | 70 (19.3) |  |
| College or above | 2782 (15.4) | 123 (19.9) |  | 2832 (15.4) | 73 (20.2) |  |
| **Paternal educational attainment, n (%)** |  |  | **<0.001** |  |  | 0.082 |
| Middle school or below | 9936 (54.9) | 296 (47.8) |  | 10,052(54.7) | 180 (49.7) |  |
| High school | 3983 (22.0) | 146 (23.6) |  | 4048 (22.0) | 81 (22.4) |  |
| College or above | 4185 (23.1) | 177 (28.6) |  | 4261 (23.2) | 101 (27.9) |  |
| **Poor sleep, n (%)** |  |  | **<0.001** |  |  | **<0.001** |
| No | 15,334 (84.7) | 324 (52.3) |  | 15,476(84.3) | 182 (50.3) |  |
| Yes | 2770 (15.3) | 295 (47.7) |  | 2885 (15.7) | 180 (49.7) |  |
| **Depressive symptoms, n (%)** |  |  | **<0.001** |  |  | **<0.001** |
| No | 14,838 (82.0) | 287 (46.4) |  | 17,233(93.9) | 241 (66.6) |  |
| Yes | 3266 (18.0) | 332 (53.6) |  | 1128 (6.1) | 121 (33.4) |  |
| **Social support score, mean(SD)** | 67.4 (15.0) | 56.4 (16.3) | **<0.001** | 67.3 (15.1) | 56.9(15.7) | **<0.001** |
| **Lifestyle risk, n (%)** |  |  | **<0.001** |  |  | **<0.001** |
| 0-1 | 3219 (17.8) | 68 (11.0) |  | 3255 (17.7) | 32 (8.8) |  |
| 2 | 8455 (46.7) | 242 (39.1) |  | 8569 (46.7) | 128 (35.4) |  |
| 3-5 | 6430 (35.5) | 309 (49.9) |  | 6537 (35.6) | 202 (55.8) |  |
| **ACEs, n (%)** |  |  | **<0.001** |  |  | **<0.001** |
| 0 | 3223 (17.8) | 32 (5.2) |  | 3241 (17.7) | 14 (3.9) |  |
| 1 | 10,046 (55.5) | 186 (30.0) |  | 10102 (55.0) | 130 (35.9) |  |
| 2-3 | 3066 (16.9) | 162 (26.2) |  | 3149 (17.2) | 79 (21.8) |  |
| 4+ | 1769 (9.8) | 239 (38.6) |  | 1869 (10.2) | 139 (38.4) |  |

Note: SD, standard deviation; ACEs, adverse childhood experiences. Categorical variables were compared using chi-square tests, and continuous variables were compared using independent-samples t tests.

**Supplementary Table S3. ACEs category descriptive statistics**

| Characteristics | ****Suicidal ideation (SI),****  **n (%)** | | **P** | ****Suicide attempt (SA),****  **n (%)** | | **P** |
| --- | --- | --- | --- | --- | --- | --- |
|  | **No** | **Yes** |  | **No** | **Yes** |  |
| **abuse** |  |  | **<0.001** |  |  | **<0.001** |
| No | 14,229 (78.6) | 279 (45.1) |  | 14,340(98.8) | 168(1.2) |  |
| Yes | 3875 (21.4) | 340 (54.9) |  | 4021(95.4) | 194(4.6) |  |
| **neglect** |  |  | **<0.001** |  |  | **<0.001** |
| No | 6439 (35.6) | 175 (28.3) |  | 6525 (35.5) | 89 (24.6) |  |
| Yes | 11,665 (64.4) | 444 (71.7) |  | 11,836 (64.5) | 273 (75.4) |  |
| **household dysfunction** |  |  | **<0.001** |  |  | **<0.001** |
| No | 14,827 (81.9) | 342 (55.3) |  | 14,970(98.7) | 199(1.3) |  |
| Yes | 3277 (18.1) | 277 (44.7) |  | 3391(95.4) | 163(4.6) |  |
| **peer/community adversity** |  |  | **<0.001** |  |  | **<0.001** |
| No | 14,786 (81.7) | 336 (54.3) |  | 14,917(98.6) | 205(1.4) |  |
| Yes | 3318 (18.3) | 283 (45.7) |  | 3444(95.6) | 157(4.4) |  |

**Supplementary Table S4. Unhealthy Lifestyle patterns descriptive statistics**

| Characteristics | ****Suicidal ideation (SI),****  **n (%)** | | **P** | ****Suicide attempt(SA),****  **n (%)** | | **P** |
| --- | --- | --- | --- | --- | --- | --- |
|  | **No** | **Yes** |  | **No** | **Yes** |  |
| **Smoking** |  |  | **<0.001** |  |  | **<0.001** |
| No | 15,962 (88.2) | 499 (80.6) |  | 16,177(98.3) | 284(1.7) |  |
| Yes | 2142 (11.8) | 120 (19.4) |  | 2184(96.6) | 78(3.4) |  |
| **Drinking** |  |  | **<0.001** |  |  | **<0.001** |
| No | 14,736 (81.4) | 420 (67.9) |  | 14,917(98.4) | 239(1.6) |  |
| Yes | 3368 (18.6) | 199 (32.1) |  | 3444(96.6) | 123(3.4) |  |
| **Inadequate physical activity** |  |  | 0.013 |  |  | 0.003 |
| No | 4695 (25.9) | 133 (21.5) |  | 4759(98.6) | 69(1.4) |  |
| Yes | 13409 (74.1) | 486 (78.5) |  | 13,602(97.9) | 293(2.1) |  |
| **Unhealthy BMI** |  |  | 0.030 |  |  | 0.053 |
| No | 12,949 (71.5) | 418 (67.5) |  | 13,125(98.2) | 242(1.8) |  |
| Yes | 5155 (28.5) | 201 (32.5) |  | 5236(97.8) | 120(2.2) |  |
| **Unhealthy diet** |  |  | **<0.001** |  |  | **<0.001** |
| No | 1417 (7.8) | 24 (3.9) |  | 1431(99.3) | 10(0.7) |  |
| Yes | 16,687 (92.2) | 595 (96.1) |  | 16,930(98.0) | 352(2.0) |  |

**Supplementary Table S5. Associations of 13 specific ACE domains with SI and SA**

| ACEs types | Categories | **aPR (95%CI)** | |
| --- | --- | --- | --- |
|  |  | **Suicidal ideation (SI)** | **Suicide attempt (SA)** |
| physical abuse | no | Reference | Reference |
|  | yes | 3.53 (3.03–4.11) | 3.51 (2.85–4.32) |
| emotional abuse | no | Reference | Reference |
|  | yes | 4.72 (4.02–5.54) | 4.30 (3.49–5.31) |
| sexual abuse | no | Reference | Reference |
|  | yes | 5.03 (3.96–6.38) | 5.03 (3.67–6.90) |
| household substance abuse | no | Reference | Reference |
|  | yes | 3.87 (2.81–5.32) | 3.79 (2.56–5.61) |
| incarcerated household member | no | Reference | Reference |
|  | yes | 2.31 (1.54–3.46) | 3.40 (2.27–5.09) |
| household mental illness | no | Reference | Reference |
|  | yes | 4.23 (3.33–5.38) | 5.20 (3.93–6.89) |
| household member  witnessing domestic violence | no | Reference | Reference |
|  | yes | 4.40 (3.73–5.19) | 4.98 (4.02–6.17) |
| parental death or divorce | no | Reference | Reference |
|  | yes | 1.88 (1.56–2.27) | 1.92 (1.49–2.46) |
| emotional neglect | no | Reference | Reference |
|  | yes | 1.33 (1.12–1.58) | 1.52 (1.21–1.93) |
| physical neglect | no | Reference | Reference |
|  | yes | 2.98 (2.11–4.20) | 3.62 (2.39–5.50) |
| bullying | no | Reference | Reference |
|  | yes | 4.31 (3.67–5.07) | 4.15 (3.33–5.18) |
| community violence | no | Reference | Reference |
|  | yes | 3.16 (2.71–3.68) | 2.76 (2.19–3.47) |
| collective violence | no | Reference | Reference |
|  | yes | 4.02 (2.96–5.47) | 3.81 (2.48–5.86) |

**Supplementary Table S6. Sensitivity analysis of joint associations and additive interactions of cumulative ACEs and unhealthy lifestyle risks with SI and SA**

| **ACEs / Lifestyle** | **Total N** | **Cases (n,%)** | **Adjusted prevalence, % (95% CI)** | **aPR (95% CI)** | **PD, percentage points (95% CI)** | **RERI (95% CI)** | **AP (95% CI)** |
| --- | --- | --- | --- | --- | --- | --- | --- |
| **Suicidal ideation (SI)** |  |  |  |  |  |  |  |
| **ACEs(0-1)** |  |  |  |  |  |  |  |
| lower lifestyle risk(0-2) | 8879 | 106 (1.2) | 1.19 (0.96–1.42) | Reference | Reference | — | — |
| higher lifestyle risk(≥3) | 4608 | 112 (2.4) | 2.42 (1.95–2.89) | 2.04 (1.50–2.57) | 1.23 (0.73–1.74) | — | — |
| **ACEs(≥2)** |  |  |  |  |  |  |  |
| lower lifestyle risk(0-2) | 3104 | 204 (6.6) | 6.66 (5.75–7.56) | 5.61 (4.30–6.92) | 5.47 (4.54–6.40) | — | — |
| higher lifestyle risk(≥3) | 2132 | 197 (9.2) | 9.40 (8.03–10.76) | 7.92 (6.06–9.77) | 8.21 (6.84–9.58) | 1.27 (-0.04–2.75) | 0.16 (-0.01–0.31) |
| **Suicide attempt (SA)** |  |  |  |  |  |  |  |
| **ACEs(0-1)** |  |  |  |  |  |  |  |
| lower lifestyle risk(0-2) | 8879 | 67 (0.8) | 0.75 (0.57–0.93) | Reference | Reference | — | — |
| higher lifestyle risk(≥3) | 4608 | 77 (1.7) | 1.67 (1.28–2.05) | 2.22 (1.52–2.92) | 0.92 (0.51–1.32) | — | — |
| **ACEs(≥2)** |  |  |  |  |  |  |  |
| lower lifestyle risk(0-2) | 3104 | 93 (3.0) | 3.03 (2.43–3.62) | 4.03 (2.79–5.26) | 2.27 (1.65–2.90) | — | — |
| higher lifestyle risk(≥3) | 2132 | 125 (5.9) | 5.92 (4.85–6.98) | 7.88 (5.59–10.17) | 5.17 (4.10–6.24) | 2.63 (0.95–4.39) | 0.33 (0.13–0.49) |

**Supplementary Table S7. Joint associations and additive interactions of specific ACE domains and lifestyle with SI and SA**

| **Specific ACEs / Lifestyle** | **Total N** | **Suicidal Ideation (SI)** | | | | **Suicide Attempts (SA)** | | | |
| --- | --- | --- | --- | --- | --- | --- | --- | --- | --- |
|  |  | **n (%)** | **aPR (95% CI)** | **RERI (95% CI)** | **AP (95% CI)** | **n (%)** | **aPR(95% CI)** | **RERI (95% CI)** | **AP (95% CI)** |
| **Abuse** |  |  |  |  |  |  |  |  |  |
| No & lower lifestyle risk(0-1) | 2627 | 36(1.3) | Reference | — | — | 17(0.6) | Reference | — | — |
| No & higher lifestyle risk(≥2) | 11881 | 243(2.0) | 1.53(1.06-2.21) | — | — | 151(1.3) | 2.02(1.21-3.35) | — | — |
| Yes & lower lifestyle risk(0-1) | 660 | 32(4.8) | 3.60(2.16-6.00) | — | — | 15(2.2) | 3.54(1.75-7.15) | — | — |
| Yes & higher lifestyle risk(≥2) | 3555 | 308(8.8) | 6.59(4.60-9.44) | 2.46(0.84-4.09) | 0.37(0.15-0.60) | 179(5.1) | 8.05(4.84-13.39) | 3.49(1.03-5.95) | 0.43(0.19-0.68) |
| **Neglect** |  |  |  |  |  |  |  |  |  |
| No & lower lifestyle risk(0-1) | 1369 | 20(1.4) | Reference | — | — | 10(0.7) | Reference | — | — |
| No & higher lifestyle risk(≥2) | 5245 | 155(2.9) | 2.09(1.30-3.38) | — | — | 79(1.5) | 2.14(1.13-4.02) | — | — |
| Yes & lower lifestyle risk(0-1) | 1918 | 48(2.5) | 1.76(1.05-2.94) | — | — | 22(1.1) | 1.62(0.76-3.56) | — | — |
| Yes & higher lifestyle risk(≥2) | 10191 | 396(3.9) | 2.78(1.76-4.40) | -0.07(-0.89-0.76) | -0.02(-0.32-0.27) | 251(2.5) | 3.54(1.89-6.61) | 0.79(-0.13-1.71) | 0.22(-0.07-0.52) |
| **Household dysfunction** |  |  |  |  |  |  |  |  |  |
| No & lower lifestyle risk(0-1) | 2726 | 46(1.7) | Reference | — | — | 24(0.9) | Reference | — | — |
| No & higher lifestyle risk(≥2) | 12443 | 296(2.4) | 1.44(1.06-1.98) | — | — | 175(1.4) | 1.64(1.08-2.49) | — | — |
| Yes & lower lifestyle risk(0-1) | 561 | 22(3.9) | 2.35(1.47-3.75) | — | — | 8(1.4) | 1.65(0.76-3.56) | — | — |
| Yes & higher lifestyle risk(≥2) | 2993 | 255(8.6) | 5.19(3.74-7.21) | 2.39(1.10-3.69) | 0.46(0.26-0.66) | 155(5.2) | 6.04(3.99-9.14) | 3.75(1.88-5.63) | 0.62(0.41-0.83) |
| **Peer/community adversity** |  |  |  |  |  |  |  |  |  |
| No & lower lifestyle risk(0-1) | 2695 | 42(1.5) | Reference | — | — | 18(0.6) | Reference | — | — |
| No & higher lifestyle risk(≥2) | 12427 | 294(2.4) | 1.56(1.12-2.17) | — | — | 187(1.5) | 2.31(1.44-3.72) | — | — |
| Yes & lower lifestyle risk(0-1) | 592 | 26(4.4) | 2.92(1.79-4.75) | — | — | 14(2.4) | 3.62(1.85-7.08) | — | — |
| Yes & higher lifestyle risk(≥2) | 3009 | 257(8.8) | 5.84(4.17-8.18) | 2.36(0.90-3.82) | 0.40(0.19-0.62) | 143(4.9) | 7.52(4.66-12.15) | 2.59(0.22-4.96) | 0.34(0.07-0.62) |

Note: ACEs, adverse childhood experiences;aPR, adjusted prevalence ratio; CI, confidence intervals; RERI, relative excess risk due to interaction; AP, attributable proportion.

Model is adjusted for sex, grade, ethnicity, residence type, sibling status, maternal educational attainment and paternal educational attainment.

**Supplementary Table S8. Sensitivity analysis including poor sleep as a sixth lifestyle-related risk factor**

| Variables | **Suicidal ideation (SI), aPR (95% CI)** | | **Suicide attempt (SA), aPR (95% CI)** | |
| --- | --- | --- | --- | --- |
|  | **Model 2** | **Model 3** | **Model 2** | **Model 3** |
| **Lifestyle-related risk Profiles** |  |  |  |  |
| 0-1(reference) | Reference | Reference | Reference | Reference |
| 2 | 1.65 (1.17–2.33) | 1.58 (1.12–2.23) | 2.24 (1.34–3.72) | 2.12 (1.28–3.53) |
| 3-6 | 3.93 (2.83–5.47) | 2.99 (2.15–4.16) | 6.13 (3.75–10.03) | 4.72 (2.89–7.71) |

Notes: ACEs, adverse childhood experiences; aPR, adjusted prevalence ratio; CI, confidence interval; PSQI, Pittsburgh Sleep Quality Index; SA, suicide attempt; SI, suicidal ideation.

Poor sleep was defined as a global PSQI score ≥8. The six-indicator lifestyle-related risk score comprised smoking, drinking, inadequate physical activity, unhealthy BMI, unhealthy diet, and poor sleep.

The cumulative score was categorized as 0–1, 2, and 3–6 risks. Model 2 was adjusted for sex, grade, ethnicity, residence type, sibling status, maternal educational attainment and paternal educational attainment. Model 3 was additionally adjusted for cumulative ACE exposure.

**Supplementary Table S9. Sensitivity analysis including poor sleep as a sixth lifestyle-related risk factor of Joint exposure to ACEs and lifestyle-related risks**

| **ACEs / Lifestyle** | **Suicidal ideation (SI), aPR (95% CI)** | **Suicide attempt (SA), aPR (95% CI)** |
| --- | --- | --- |
| **ACEs(0-3)** |  |  |
| lower lifestyle-related risk(0-1) | Reference | Reference |
| higher lifestyle-related risks(≥2) | 2.13 (1.48–3.08) | 3.04 (1.80–5.13) |
| **ACEs(≥4)** |  |  |
| lower lifestyle-related risk(0-1) | 2.76 (1.24–6.12) | 1.85 (0.42–8.14) |
| higher lifestyle-related risks(≥2) | **11.40 (7.82–16.62)** | 15.78 (9.29–26.83) |

Notes: ACEs, adverse childhood experiences; aPR, adjusted prevalence ratio; CI, confidence interval; SA, suicide attempt; SI, suicidal ideation. The joint-exposure model compared combinations of ACE exposure (<4 vs. ≥4 ACEs) and lifestyle-related risks (0–1 vs. ≥2 risks), with <4 ACEs and 0–1 lifestyle-related risks as the reference group

**Supplementary Table S10. Abbreviations used in tables and figures**

| Abbreviation | Full term |
| --- | --- |
| SI | suicidal ideation |
| SA | suicide attempt |
| ACEs | adverse childhood experiences |
| PhA | physical abuse |
| EmA | emotional abuse |
| SeA | sexual abuse |
| HSA | household substance abuse |
| IHM | incarcerated household member |
| HMI | household mental illness |
| HMW | household member witnessing domestic violence |
| PDD | parental death or divorce |
| EmN | emotional neglect |
| PhN | physical neglect |
| BUL | bullying |
| CMV | community violence |
| CLV | collective violence |
| SMK | smoking |
| DRK | drinking |
| UAc | inadequate physical activity |
| BMI | unhealthy BMI |
| UDi | unhealthy diet |
| SEX | sex |
| GRD | grade |
| RAC | ethnicity |
| ADD | residence type |
| MED | maternal education |
| FED | paternal education |
| DEP | depressive symptoms |
| SLP | poor sleep |
| SOC | social support |
| ONL | only child/ sibling status |

**Supplementary Figure S1. Network structure of suicidal ideation, adverse childhood experiences, and unhealthy lifestyle factors.**

Panel A shows the network before controlling for covariates, with suicidal ideation (SI), overall adverse childhood experiences (ACEs), and lifestyle (LIF) variables. Panel B shows the corresponding network after controlling for sociodemographic characteristics, depressive symptoms, poor sleep, and social support. Panels C and D present networks using the 13 individual ACE domains and five lifestyle factors before and after covariate adjustment, respectively.

**Supplementary Figure S2. Network structure of suicide attempt, adverse childhood experiences, and unhealthy lifestyle factors.**

Panel A shows the network before controlling for covariates, including suicide attempt (SA), overall adverse childhood experiences (ACEs), and lifestyle (LIF) variables. Panel B shows the corresponding network after controlling for sociodemographic characteristics, depressive symptoms, poor sleep, and social support. Panels C and D present networks including the 13 individual ACE domains and five lifestyle factors before and after covariate adjustment,respectively.

**Supplementary Figure S3. Bootstrap stability and edge-weight accuracy of the adjusted dimension-level networks.**

Panels A and B show case-dropping bootstrap analyses of expected influence in the SI and SA networks, respectively. Panels C and D show nonparametric bootstrap confidence interval for estimated edge weights. Correlation stability coefficients were calculated using case-dropping bootstrap procedures.
